# Diagnostic value of decoy receptor 3 combined with procalcitonin and soluble urokinase-type plasminogen activator receptor for sepsis

**DOI:** 10.1186/s11658-018-0087-z

**Published:** 2018-05-09

**Authors:** Jing-jing Zhao, Xiao-Li Lou, Hong-wei Chen, Feng-ting Zhu, Yan-Qiang Hou

**Affiliations:** Department of Central Laboratory, Songjiang Hospital Affiliated First People’s Hospital, Shanghai Jiao Tong University, Shanghai, 201600 China

**Keywords:** Sepsis, SIRS, DcR3, suPAR, PCT

## Abstract

The levels of decoy receptor 3 (DcR3), soluble urokinase type plasminogen activator receptor (suPAR) and procalcitonin (PCT) are significantly increased in sepsis. We investigated the diagnostic value of DcR3 combined with suPAR and PCT in sepsis. Patients with sepsis, non-infectious systemic inflammatory response comprehensive syndrome (SIRS) and healthy controls were recruited according to the diagnostic standard. We measured DcR3, suPAR, PCT, interleukin-6 (IL-6) and C-reactive protein (CRP), and the diagnostic value was evaluated by receiver operating characteristics (ROC) curves. In our analysis, serum DcR3, suPAR and PCT levels of the sepsis group were significantly higher than those of the SIRS and control groups. However, IL-6, CRP and WBC showed no significant difference between the SIRS group and the sepsis group. The serum DcR3 level was positively correlated with the serum suPAR level (*r* = 0.37, *p* = 0.0022) and PCT level (*r* = 0.37, *p* = 0.0021). Using DcR3, suPAR and PCT to distinguish SIRS from sepsis, the area under the curve (AUC) values were 0.892, 0.778 and 0.692. When DcR3, suPAR and PCT combined were used for diagnosis of sepsis, the AUC was 0.933, at a cut-off point of 0.342. This combination improved the sensitivity and specificity of diagnosis of sepsis, suggesting that use of the combination of three indexes enhanced the efficiency of sepsis diagnosis.

## Introduction

Sepsis is a systemic inflammatory response syndrome (SIRS) caused by infection that eventually leads to organ dysfunction and death. SIRS is the leading cause of death in intensive care unit (ICU) patients. According to the global estimates, there are 31.5 million sepsis and 19.4 million severe sepsis cases, with potentially 5.3 million deaths annually. The mortality rate of sepsis decreases year by year, but the number of patients who die from sepsis is increasing with increasing morbidity [1, 2]. The identification of causative pathogens through blood cultures is still the gold standard of sepsis diagnosis. However, confirmation of pathogens by cultures is slow and it often yields false negative results [3]. The problem of effectively treating patients with sepsis is in part attributed to the difficulty of accurately diagnosing sepsis especially in its early stages. Therefore, better and faster biological indicators in the early diagnosis of sepsis are sought.

Decoy receptor 3 (DcR3) is a new member of the tumor necrosis factor receptors superfamily, which competitively binds Fas ligand, LIGHT and TL1A to block apoptosis. Previous studies have suggested that DcR3 expression is upregulated in inflammatory diseases, such as bacterial infections, rheumatoid arthritis, acute ulcerative colitis, appendicitis, and cancers, and primarily functions to prevent inflammation and apoptosis [4, 5]. Our preliminary study showed that DcR3 expression has diagnostic value for sepsis [6], and is a valuable marker to predict the outcome of sepsis [7].

Currently, procalcitonin (PCT) and soluble urokinase type plasminogen activator receptor (suPAR) are used as laboratory diagnostic indicators of sepsis. While these tests have a wide range of clinical applications, better tests would be helpful for patients [8–11]. This study aimed to explore the value of measuring DcR3 combined with suPAR and PCT expression in the clinical diagnosis of sepsis.

## Materials and methods

### Study subjects

Thirty-four patients with sepsis, who were hospitalized in the intensive care unit of our hospital in Songjiang District Center Hospital, Shanghai, China, from August 2015 to December 2016, were recruited. Meanwhile, 34 patients with SIRS and 20 healthy subjects who had not recently suffered from infection or autoimmune disease and with normal results obtained by physical examination were recruited as healthy controls.

Inclusion criteria: age ≥ 18 years, sepsis diagnosis conforms to the standard of the Surviving Sepsis Campaign: International Guidelines for Management of Severe Sepsis and Septic Shock [12]. Patients whose SIRS diagnosis conformed to the standard of the 1991 ACCP/SCCM were also recruited [13].

Exclusion criteria: (1) HIV infection, confirmed rheumatoid arthritis, white blood cell (WBC) count < 1 × 10^9^/L or neutrophil cells count < 0.5 × 10^9^/L; (2) onset of acute myocardial infarction, cerebral infarction or hemorrhage in the past 6 months; (3) comorbidity of severe liver, kidney malfunction, heart failure, blood disease, malignant tumor, and psychiatric patients; (4) use of glucocorticoids equal to a dosage of 1 mg/kg of prednisone for > 1 month, use of immunosuppressive drugs, or death within 24 h of enrollment in the intensive care unit.

### Collection of blood samples and measurement of indicators

Venous blood samples (2 mL) were immediately collected from sepsis and SIRS patients before treatment when they were admitted to the hospital. Blood was harvested in test tubes with coagulant and subjected to mixing and centrifugation at 3500 rpm for 15 min at 4 °C. Serum was immediately separated, transferred into frozen tubes and stored at − 80 °C. Serum of the control group was collected in the same way. Serum DcR3 (RayBio, Norcross, GA, USA) and serum suPAR (ViroGates, Birkerod, Denmark) levels were detected by an human enzyme-linked immunosorbent assay (ELISA) kit using an iMark Microplate reader (Bio-Rad, Hercules, CA, USA); interleukin-6 (IL-6) and PCT levels were determined using electrochemical luminescence with a Cobas e601 instrument (Roche, Basle, Switzerland).

### Statistical methods

SPSS version 19.0 (SPSS Inc., Chicago, IL, USA) software was employed to carry out the statistical analysis. The Mann-Whitney U test was used to compare continuous parametric variables. One-way analysis of variance or the Kruskal-Wallis test was used to compare more than two groups of quantitative data. A 2-sided *P* < 0.05 was considered significant. Spearman’s rank correlation analysis was used to determine bi-variant relationships. Receiver operating characteristics (ROC) curves were constructed and the area under the curve (AUC) was calculated. Significant indexes were combined as joint diagnostic indexes. Logistic regression analysis was used to construct combined predictors. All data are presented as mean ± standard error or median (minimum–maximum).

## Results

### General information and clinical data of the patients

We found that there was no statistically significant difference (*P* > 0.05) in age and sex among the sepsis group (*n* = 34), SIRS group (*n* = 34) and control group (*n* = 20). The IL-6, WBC and C-reactive protein (CRP) levels of the sepsis group and SIRS group were significantly higher than those of the control group (*P* < 0.05). However, there was no significant difference between the SIRS group and sepsis group (Table 1).

**Table 1:** Clinical data of patients and healthy controls

	Control (*n* = 20)	SIRS (*n* = 34)	Sepsis (*n* = 34)
Age	63.20 ± 8.82	57.06 ± 21.91	67.50 ± 12.59
Gender (M/F)	14/6	20/14	20/14
IL-6 (pg/mL)	6.63 (3.42–23.39)	77.41 (3.31–1971.00)**	100.65 (8.41–5900.00)**
WBC (×10^9^/L)	6.87 ± 1.62	16.62 ± 4.58**	18.40 ± 3.52**
CRP (mg/L)	4.00 (1.00–33.00)	37.50 (1.00–177.00)**	51.00 (0.49–325.00)**

compared with control, **p < 0.01

### Change of serum DcR3, suPAR and PCT levels in patients with sepsis

**Fig. 1:**
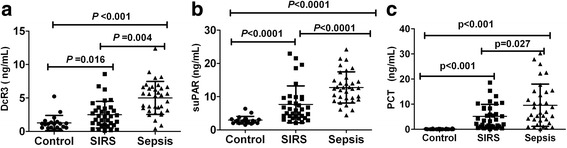
Levels of DcR3, suPAR and PCT **a**: Minimum-maximum (median) value of serum DcR3 in sepsis (*n* = 34), SIRS (*n* = 34) and controls (*n* = 20) by non-parametric Kruskal–Wallis test (*p* < 0.001). **b**: mean ± SD value of serum suPAR in controls (*n* = 20), SIRS (*n* = 34) and sepsis (*n* = 34), respectively, by Mann–Whitney test (*p* < 0.001). **c**: Minimum-maximum (median) value of PCT in controls (*n* = 20), SIRS (*n* = 34) and sepsis (*n* = 34), by all non-parametric Kruskal–Wallis test (*p* < 0.001)

The serum DcR3 level of the sepsis group [4.65 (0.38, 27.62) ng/mL] was significantly higher than that of the SIRS group [0.58 (0.00, 8.72) ng/mL] and control group [0.16 (0.00, 0.73 ng/mL] (both *P* < 0.001; Fig. 1a). The serum suPAR level of the sepsis group (12.78 ± 5.19 ng/mL) was also significantly higher than that of the SIRS group (7.67 ± 5.56 ng/mL) and control group (2.93 ± 1.14 ng/mL) (*P* < 0.001; Fig. 1b). The serum PCT level of the sepsis group [6.58 (0.13, 30.25) ng/mL] was significantly higher than that of the SIRS [1.39 (0.03, 20.28) ng/mL] (*P* < 0.001) and control group [0.065 (0.02, 0.75) ng/mL] (*P* < 0.05; Fig. 1c).

### Correlation of DcR3 levels with other indicators

**Fig. 2:**
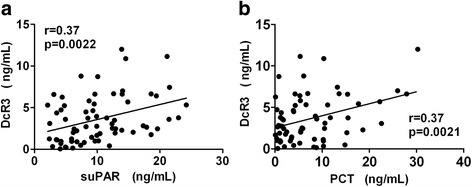
Correlation between DcR3 and PCT (**a**, *r* = 0.37, *p* = 0.0021), DcR3 and suPAR (**b**, *r* = 0.37, *p* = 0.0022), respectively

To determine the correlation between DcR3 levels and other indicators, the indexes of the sepsis group were subjected to correlation analysis. Serum DcR3 was positively correlated with PCT and suPAR (Fig. 2).

### Diagnostic value of single indicator and combination for distinguishing control group from SIRS

**Fig. 3:**
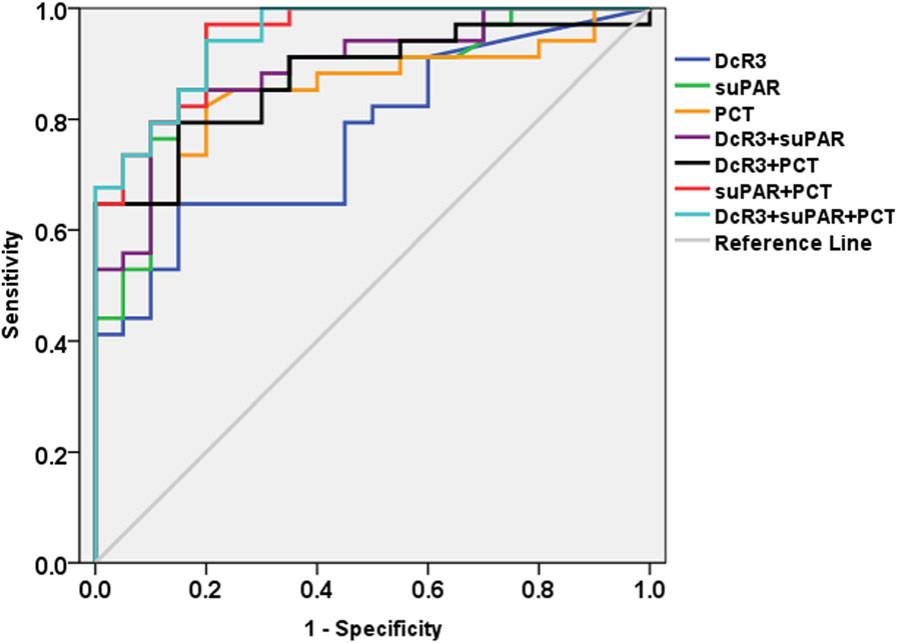
ROC evaluation of DcR3, suPAR, PCT and DcR3 combined with suPAR, PCT in control group vs. SIRS group. The ROC evaluation was performed at cut-off values recommended by the scientific community of laboratory medicine with 95% CI

The predictive values of the indicators for SIRS were determined and compared. Compared with the control group, the AUC values of DcR3, suPAR, and PCT were 0.768, 0.876 and 0.858, respectively (Fig. 3). The sensitivity and specificity of suPAR and PCT for SIRS were higher than those of DcR3. The AUC values of the combinations of DcR3 + suPAR, DcR3 + PCT, suPAR+PCT, DcR3 + suPAR+PCT were 0.893, 0.875, 0.946 and 0.947; the sensitivity values were 85.3, 79.4, 97.1 and 94.1%; the specificity values were 85.0, 85.0, 80.0 and 80.0%, respectively. It suggested that the co-detection of DcR3 + suPAR+PCT for SIRS did not improve the diagnostic efficiency (Table 2).

**Table 2:** Receiver operating characteristic analysis of DcR3, suPAR and PCT in control and SIRS

	AUC	SID error	Sig	95%CI		Cut-off value	Sensitivity	Specificity
				Lower limit	Upper limit			
DcR3	0.768	0.063	0.001	0.643	0.892	0.190 ng/mL	0.79.4	0.550
suPAR	0.876	0.048	0.000	0.781	0.970	0.519 ng/mL	0.85.3	0.850
PCT	0.858	0.051	0.000	0.758	0.958	0.375 ng/mL	0.824	0.800
DcR3 + suPAR	0.893	0.044	0.000	0.807	0.978	0.440	0.853	0.850
DcR3 + PCT	0.875	0.047	0.000	0.783	0.967	0.517	0.794	0.850
suPAR+PCT	0.946	0.029	0.000	0.890	1.000	0.308	0.971	0.800
DcR3 + suPAR+PCT	0.947	0.028	0.000	0.893	1.000	0.310	0.941	0.800

### Diagnostic value of single indicator and combination for distinguishing control group from sepsis

**Fig. 4:**
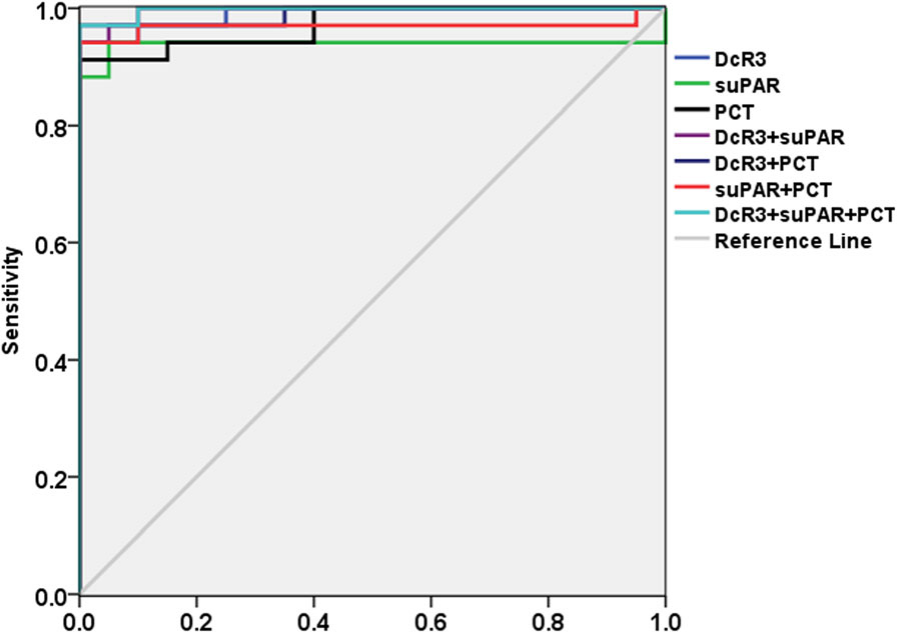
ROC evaluation of DcR3, suPAR, PCT and DcR3 combined with suPAR, PCT in control group vs. sepsis group. The ROC evaluation was performed at cut-off values recommended by the scientific community of laboratory medicine with 95% CI

DcR3, suPAR and PCT were evaluated by ROC curve analysis for the control group vs. the sepsis group (Fig. 4), with the AUC determined to be 0.990, 0.938 and 0.972, respectively. The AUC of DcR3 was found to be greater than that of suPAR and PCT, suggesting that DcR3 was better than suPAR and PCT in the diagnosis of sepsis. The AUC of DcR3 + suPAR+PCT is 0.997, which is better than DcR3 + suPAR (AUC = 0.996) or DcR3 + PCT (AUC = 0.990) or suPAR+PCT (AUC = 0.969), and DcR3 + suPAR+PCT has the best specificity, indicating that the combination of the three indexes had the best diagnostic performance in sepsis (Table 3).

**Table 3:** Receiver operating characteristic analysis of DcR3, suPAR and PCT in control and sepsis

	AUC	SID error	Sig	95%CI		Cut-off value	Sensitivity	Specificity
				Lower limit	Upper limit			
DcR3	0.990	0.009	0.000	0.971	1.000	0.545 ng/mL	0.971	0.900
suPAR	0.938	0.040	0.000	0.859	1.000	5.535 ng/mL	0.941	0.850
PCT	0.972	0.019	0.000	0.935	1.000	0.513 ng/mL	0.941	0.870
DcR3 + suPAR	0.996	0.005	0.000	0.986	1.000	0.489	0.971	0.950
DcR3 + PCT	0.990	0.011	0.000	0.968	1.000	0.237	0.971	0.950
suPAR + PCT	0.969	0.028	0.000	0.915	1.000	0.297	0.971	0.900
DcR3 + suPAR + PCT	0.997	0.004	0.000	0.989	1.000	0.345	0.971	0.980

### Diagnostic value of single indicator and combination for distinguishing SIRS from sepsis

**Fig. 5:**
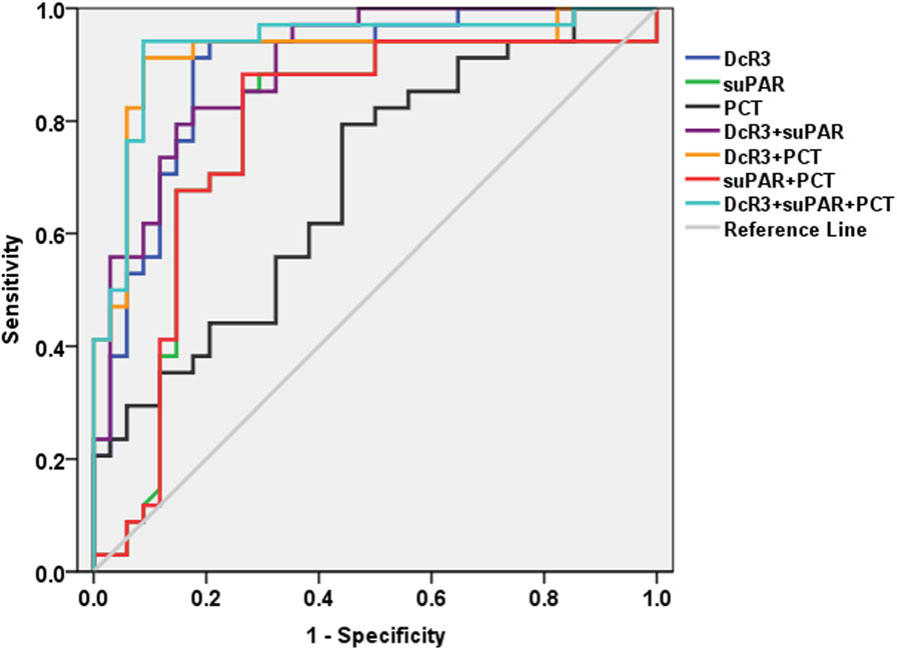
ROC evaluation of DcR3, suPAR, PCT and DcR3 combined with suPAR, PCT in SIRS group vs. sepsis group. The ROC evaluation was performed at cut-off values recommended by the scientific community of laboratory medicine with 95% CI

In order to further clarify the diagnostic value, DcR3, suPAR and PCT were used to distinguish SIRS from sepsis. Compared with the SIRS group, the AUC of DcR3 was the best predictor for sepsis (0.892 vs. 0.778 (suPAR) vs. 0.692 (PCT), respectively). When the DcR3 level was 1.690 ng/mL, the sensitivity and specificity were 91.2 and 82.4%, indicating that DcR3 has potential application value in the diagnosis of sepsis. The AUC of DcR3 + suPAR+PCT combined was 0.933, higher than for DcR3 + suPAR (AUC = 0.897) or DcR3 + PCT (AUC = 0.916) or suPAR+PCT (AUC = 0.779). When the cut-off of DcR3 + suPAR+PCT was 0.342, the sensitivity and specificity were 94.1 and 91.2%. (Fig. 5) Thus, the combination of the three indexes enhanced the accuracy and prediction efficiency, compared to a single index (Table 4).

**Table 4:** Receiver operating characteristic analysis of DcR3, suPAR and PCT in SIRS and sepsis

	AUC	SID error	Sig	95%CI		Cut-off value	Sensitivity	Specificity
				Lower limit	Upper limit			
DcR3	0.892	0.040	0.000	0.813	0.971	1.690 ng/mL	0.912	0.824
suPAR	0.778	0.062	0.000	0.657	0.900	8.355 ng/mL	0.853	0.735
PCT	0.692	0.064	0.006	0.568	0.817	2.255 ng/mL	0.794	0.559
DcR3 + suPAR	0.897	0.037	0.000	0.824	0.970	0.374	0.824	0.824
DcR3 + PCT	0.916	0.039	0.000	0.840	0.993	0.356	0.912	0.912
suPAR + PCT	0.779	0.062	0.000	0.658	0.901	0.427	0.882	0.735
DcR3 + suPAR + PCT	0.933	0.034	0.000	0.867	0.998	0.342	0.941	0.912

## Discussion

Sepsis is a rapidly propagating excessive inflammatory reaction that occurs in response to a variety of pathogenic bacteria entering the blood system, producing a large number of toxins. The laboratory indicators used for the clinical diagnosis of sepsis include CRP, IL-6, IL-8, tumor necrosis factor-α (TNF-α) and PCT, none of which are ideal diagnostic indicators due to their deficiencies in diagnostic specificity and sensitivity [14–17]. For many years, researchers have carried out a large number of studies and clinical trials, but the pathogenesis of sepsis is still not fully elucidated, which also hampers effective treatment, resulting in high mortality [18, 19]. Therefore, it is important to find a new diagnostic indicator to improve the treatment of sepsis and reduce mortality.

Currently, PCT is considered to be the best laboratory diagnostic indicator of sepsis, but its value for diagnosis, prognosis and the differential diagnosis with SIRS is not satisfactory [20–22]. A meta-analysis showed that the sensitivity and specificity of PCT in early sepsis diagnosis in critically ill patients were 77% (95% CI: 72–81%) and 79% (95% CI: 74–84%). However, since PCT levels can rise due to surgery as well as inflammatory and autoimmune diseases, its specificity in sepsis diagnosis is limited [23]. Pierrakos et al. [24] analyzed 178 diagnostic indicators of sepsis in 3370 papers, concluding that PCT is deficient for diagnosis, differential diagnosis and prognosis of sepsis. In this study, the AUC, sensitivity and specificity of PCT were determined to be 0.692, 79.4 and 55.9%, respectively, which also indicated that the diagnostic specificity of PCT was low in sepsis diagnosis. Therefore, it is urgent to find new indicators for the diagnosis and prognosis of sepsis.

SuPAR is the soluble form of urokinase type plasminogen activator receptor (uPAR). Under inflammatory stimulation, uPAR is removed from the cell surface through the activity of a variety of proteases, forming suPAR [25]. Increased suPAR levels, which were believed to be a good biomarker for sepsis diagnosis, primarily occur in patients with cancer and a variety of infectious and inflammatory diseases [26]. Recent studies have shown that suPAR levels are significantly increased in sepsis and could reflect the severity of sepsis [27–29]. *In vivo* studies have found that stimulation by high dose endotoxin can increase suPAR levels, while low dose endotoxin stimulation failed to increase suPAR levels [30]. This study found that suPAR levels were significantly increased in sepsis patients. When compared with the control group, at a cut-off point of 5.535 ng/mL, the sensitivity and specificity for diagnosis of sepsis using suPAR were 94.1 and 85.0%, and the PCT was comparable, while the area under the ROC curve was smaller than the PCT, similar to the results of the study published by Zeng [31]. When distinguishing SIRS from sepsis using suPAR, the optimal cut-off point was 8.355 ng/mL. At this point, the sensitivity and specificity were 85.3 and 73.5%, respectively, which was better than PCT, indicating that suPAR had a better sensitivity in sepsis diagnosis.

DcR3 is a member of the soluble tumor necrosis factor receptor superfamily lacking transmembrane structures. Some studies have shown that DcR3 can reduce inflammatory responses by promoting the secretion of anti-inflammatory factors and down-regulating the expression of inflammatory factors [32, 33]. Our previous study established a mouse model of sepsis and applied dose-dependent DcR3 treatment. It showed that DcR3 significantly inhibited the inflammatory reaction, and reduced lymphocyte apoptosis in the thymus and spleen, improving survival rates [34]. DcR3 can modulate macrophage differentiation and the secretion of inflammatory cytokines and chemokines, functioning as part of the immune surveillance and immune regulation systems, indicating that DcR3 may play a role in the early pathological mechanisms of sepsis [35]. In this study, we found that the DcR3 level was significantly increased in sepsis patients. Furthermore, on evaluation of the ROC curve, our findings showed that when the DcR3 cut-off point was 1.690 ng/mL, the sensitivity and specificity of DcR3 were 91.2 and 82.4%, which was better than those of suPAR and PCT to distinguish SIRS from sepsis, indicating that DcR3 shows great promise for use as a diagnostic biomarker of sepsis. Gao et al. [36] found that DcR3 increased significantly in the early stage of sepsis and monitoring its outcome, especially when sepsis patients were PCT negative. However, DcR3 levels showed no difference among various pathogens associated with sepsis.

Although the above three biological indicators have certain application value in the early diagnosis of sepsis, they are limited by various conditions and cannot be used independently as an ideal indicator for diagnosis of sepsis. Therefore, in order to improve the early diagnosis of sepsis, we also evaluated the diagnostic value of combined examination of DcR3, suPAR and PCT by the ROC curve. Our results suggested that the effect of a single indicator in the diagnosis of sepsis is not ideal, and diagnosis using multiple indicators in combination may be more effective [24]. Compared with the control group, the sensitivity and specificity of DcR3 + suPAR+PCT for sepsis were 97.1 and 98.0%, which were superior to those of suPAR+PCT or DcR3 + suPAR or DcR3 + PCT. DcR3, suPAR and PCT were used to distinguish SIRS from sepsis; when the optimal cut-off point of DcR3 + suPAR+PCT was 0.342, the sensitivity and specificity were 94.1 and 91.2%, showing that this combination enhanced the accuracy and prediction efficiency, compared to a single index. Moreover, in this study a correlation analysis was carried out among DcR3, suPAR and PCT, which showed that DcR3 was correlated with suPAR and PCT, suggesting that use of the combination of the three indexes has a higher clinical diagnostic value for sepsis.

In summary, the host response to sepsis involves hundreds of mediators and single molecules, many of which have been proposed to be sepsis biomarkers. It is unlikely that is able to satisfy all the existing needs and expectations in sepsis research and management. The combined diagnostic value of the three indicators is higher than that of the single indicator. However, the number of samples in this study was small, and further large-scale clinical studies are needed to verify the results, and to provide new ideas for the pathogenesis of sepsis and early treatment.

## Abbreviations

ACCP/SCCM: American College of Clinical Pharmacy/Society of Critical Care Medicine
HIV: Human immunodeficiency virus
LIGHT: A receptor expressed by T lymphocytes
TL1A: TNF-like ligand 1A
